# Do Web-Based and Clinic Samples of Gay Men Living With HIV Differ on Self-Reported Physical and Psychological Symptoms? A Comparative Analysis

**DOI:** 10.2196/jmir.3800

**Published:** 2015-03-19

**Authors:** Richard Harding, Fiona Lampe, Tim Molloy, Lorraine Sherr

**Affiliations:** ^1^Cicely Saunders InstituteDepartment of Palliative Care, Policy & RehabilitationKing\\\'s College LondonLondonUnited Kingdom; ^2^University College LondonDepartment of Infection and Population HealthLondonUnited Kingdom; ^3^GMFALondonUnited Kingdom

**Keywords:** HIV, pain, symptoms, mental health, methods, recruitment, sampling, Internet

## Abstract

**Background:**

Although the Internet is commonly used to recruit samples in studies of human immunodeficiency virus (HIV)-related risk behaviors, it has not been used to measure patient-reported well-being. As the burden of long-term chronic HIV infection rises, the Internet may offer enormous potential for recruitment to research and interventions.

**Objective:**

This study aimed to compare two samples of gay men living with HIV, one recruited via the Web and the other recruited in outpatient settings, in terms of self-reported physical and psychological symptom burden.

**Methods:**

The Internet sample was recruited from a UK-wide Web-based survey of gay men with diagnosed HIV. Of these, 154 respondents identified themselves as resident in London and were included in this analysis. The HIV clinic sample was recruited from five HIV outpatient clinics. Of these participants, 400 gay men recruited in London clinics were included in this analysis.

**Results:**

The Web-based sample was younger than the clinic sample (37.3 years, SD 7.0 vs 40.9 years, SD 8.3), more likely to be in paid employment (72.8%, 99/136 vs 60.1%, 227/378), less likely to be on antiretroviral therapy (ART) (58.4%, 90/154 vs 68.0%, 266/391), and had worse mean psychological symptom burden compared to the clinic sample (mean scores: 1.61, SD 1.09 vs 1.36, SD 0.96) but similar physical symptom burden (mean scores: 0.78, SD 0.65 vs 0.70, SD 0.74). In multivariable logistic regression, for the physical symptom burden model, adjusted for age, ethnicity, employment status, and ART use, the recruitment setting (ie, Web-based vs clinic) was not significantly associated with high physical symptom score. The only variable that remained significantly associated with high physical symptom score was employment status, with those in employment being less likely to report being in the upper (worst) physical symptom tertile versus the other two tertiles (adjusted OR 0.41, 95% CI 0.28-0.62, *P*<.001). For the psychological symptom burden model, those recruited via the Web were significantly more likely to report being in the upper (worst) tertile (adjusted OR 2.20, 95% CI 1.41-3.44, *P*=.001). In addition, those in employment were less likely to report being in the upper (worst) psychological symptom tertile compared to those not in employment (adjusted OR 0.32, 95% CI 0.21-0.49, *P*<.001).

**Conclusions:**

Our data have revealed a number of differences. Compared to the clinic sample, the Web-based sample had worse psychological symptom burden, younger average age, higher prevalence of employment, and a lower proportion on ART. For future research, we recommend that Web-based data collection should include the demographic variables that we note differed between samples. In addition, we recognize that each recruitment method may bring inherent sampling bias, with clinic populations differing by geographical location and reflecting those accessing regular medical care, and Web-based sampling recruiting those with greater Internet access and identifying survey materials through specific searches and contact with specific websites.

##  Introduction

Research protocols that utilize electronic and Web-based methods of participant recruitment to research and intervention participation and associated data collection activities have become increasingly common. The method has become particularly well used in behavioral surveillance research studies among persons living with human immunodeficiency virus (HIV) infection in high-income countries. Web-based methods have been used in various ways in epidemiological HIV studies investigating risk behavior [1], to conduct Web-based interventions [2], and to determine the role of the Internet as a phenomenon itself in sexual behavior [3].

A review of the methodological implications of Web-based HIV behavioral surveillance methods summarized the advantages as convenience, reduced costs in the management of tools and data collection and entry, ease of tool modification, anonymity, and reduced social desirability bias [4]. The identified disadvantages were the impossibility of implementing a sampling frame, the potential for limited generalizability due to convenience and self-selected sampling, the challenges of calculating a response rate, and the biases of access to (and familiarity with) Internet use.

A review of HIV behavioral research among men who have sex with men (MSM) found equivocal evidence in the literature as to whether men who use the Internet are more likely to report risk behavior, although those who use the Internet for sex are more likely to be younger, report sex with women, have had a sexually transmitted infection, and to use a public sex environment [5]. The specific websites used for recruitment may also introduce sampling bias, with different demographic profiles between sites [6].

A comparison of London MSM recruited via the Web and in the community found that the Web-based sample were younger and were less likely to exclusively have sex with men, to be in a relationship with a man, to have received higher education, or to have been tested for HIV [7]. A further study comparing a sample of UK-wide MSM recruited online to a second study that conducted a national random probability population sample found no significant differences in reported ethnicity, education, social class, country of birth, alcohol consumption, injecting drug use, or age of first male sexual partner. However, there was strong evidence that men in the Internet sample were younger, less likely to live in London, less likely to report being in good health, and more likely to be working [8]. The evidence to date has been of MSM and the general population, but it has not addressed the specific characteristics of people with diagnosed HIV.

There have been methodological advances in the design and implementation of Web-based recruitment and data collection to investigate behavioral aspects of HIV infection (principally primary prevention and risk behavior). However, the utility of using these methods to investigate disease-oriented variables and patient self-report burden of disease has not been explored. As much health research and delivery becomes oriented to long-term and chronic conditions (where the patient-reported experience is an important area of inquiry, and those living with long-term chronic conditions have greater potential to use the Internet as compared to rapidly declining conditions), there may be great potential for use of the Internet for recruitment and data collection for both research and care activities. The rise in the use of patient-reported outcome measures (PROMS) to improve equity and quality in health care [9] gives great potential for Web-based self-reporting of health states. Self-reported physical and psychological symptom prevalence and intensity is an important area of clinical HIV research, as recent evidence has shown that symptoms are associated with sexual risk-taking (with higher psychological symptom burden associated with risk taking) [10], with poor adherence to antiretroviral treatment (associated with higher psychological and global symptom burden) [11], treatment switching (associated with higher psychological and physical symptom burden) [12], viral rebound (predicted by higher physical, psychological and global symptom burden) [13], poorer quality of life (associated with higher physical and psychological burden) [14], and suicidal ideation (associated with higher physical and psychological burden) [15].

This study aimed to compare two samples of gay men living with HIV, one recruited via the Web and the other recruited in HIV outpatient settings, in terms of self-report physical and psychological symptom burden. The outcome of interest was the self-report 7-day period prevalence and burden of physical and psychological symptoms.

## Methods

### Overview

The study is a secondary analysis of two datasets (one recruited via the Web and one in outpatient clinics). Participants were gay men with diagnosed HIV who were resident in London, United Kingdom.

### Settings and Recruitment Methods

We summarize the two prior study designs here. The Web-based sample [16] was recruited through banners on gay-interest and health information websites for a UK-wide Web-based survey of men who identified as gay with diagnosed HIV (N=347). Of these, 154 respondents identified themselves as resident in London and were included in this analysis.

The HIV clinic sample [15] was recruited in a cross-sectional study conducted in five HIV outpatient clinics in London and the south east of England. Consecutive HIV-diagnosed attending patients were approached and invited to participate by filling out a self-completed questionnaire, and 778 were completed and returned (77%, 778/1010 of all patients; 86.0%, 778/905 of those eligible to receive a questionnaire). Of the participants, 400 men who identified as gay recruited in London clinics were included in this analysis. All data were anonymized, and no identifiable information was available during data merging.

### Common Questionnaire Items Between Samples

In both studies, symptoms were measured using the Memorial Symptom Assessment Scale−Short Form (MSAS-SF), a patient self-report scale that measures the 7-day period prevalence of 26 physical and 6 psychological symptoms. This standardized symptom questionnaire captures the presence of each symptom and associated distress (for physical symptoms) or frequency (for psychological symptoms) and has often been reported in studies of people living with HIV [17-20]. Three summary subscale indices can be derived: Physical Symptom Distress (MSAS-Phys), Psychological Symptom Distress (MSAS-Psych), and Global Distress Index (MSAS-GDI) [14]. Each of these 3 subscales has a possible score range of 0-4.

Respondents in both studies gave demographic data on age (analyzed as a continuous variable), ethnicity (categorized as white/non-white), education (categorized as university/non-university), employment (categorized as currently in paid employment or not), and current antiretroviral therapy (ART) use (yes/no).

### Analysis

For each sample (clinic and Web-based), we present descriptive analyses for the demographic characteristics and MSAS-SF variables (total number of symptoms, global distress subscale, physical distress subscale, and psychological distress subscale). The demographic variables were compared between samples (clinic vs Web-based) using *t* tests for continuous variables, and chi-square tests for categorical variables. The symptom subscales were compared between samples using *t* tests; additionally, the subscales were categorized into tertiles and compared between samples using chi-square test for trend. Subsequently, in light of the associations between demographic characteristics and sample source (ie, Web-based vs not Web-based), we constructed two multivariable logistic regression models with the MSAS-SF physical and psychological symptom scores as the dependent variables, categorized as the upper (worst) tertile of each score versus the remaining two tertiles. These models aimed to determine whether sample source is independently associated with poorer physical and psychological outcomes when adjusting for demographic factors. Variables significant at the 10% level (*P*<.10) in univariate analysis were entered into the multivariable logistic models [20]. Results from these models are presented as odds ratios (OR) with 95% confidence intervals (CI).

## Results

### Sample Comparison

The comparison of characteristics between the two samples is presented in Table 1, demonstrating the statistically significant differences identified between samples. The Web-based sample was younger, more likely to be in paid employment, less likely to be on ART, and had worse mean psychological symptom burden compared to the clinic sample. The test for trend revealed that the Web-based sample had a comparatively lower proportion in the worst tertile for physical symptoms, but a higher proportion in the worst psychological tertile, compared to the clinic group.

The multivariable analysis assessed the effect of setting on symptom score (predicting having a score in the highest tertile compared to the other two) after adjusting for all factors with *P*<.1 in univariable analysis (ie, age, ethnicity, employment, ART use). For the physical symptom burden model, the recruitment setting (ie, clinic vs Web-based) was not significant after adjusting for the other factors. The only variable that remained significantly associated with high physical symptom score was employment status, with those in employment being less likely to report being in the upper (worst) physical symptom tertile (adjusted OR 0.41, 95% CI 0.28-0.62, *P*<.001). For the psychological symptom burden model, those recruited via the Web were significantly more likely to report being in the upper (worst) tertiles (adjusted OR 2.20, 95% CI 1.41-3.44, *P*=.001) compared to the clinic sample. In addition, those in employment were less likely to report being in the upper (worst) psychological symptom tertile compared to those not in employment (adjusted OR 0.32, 95% CI 0.21-0.49, *P*<.001). See Table 2.

**Table 1:** Univariate comparison of the clinic versus Web-based samples.

			Clinic setting (n=400)	Web-based setting (n=154)	Test comparison	Degrees of freedom
**Age in years, mean (SD) median**						
	Missing: clinic n=6; Web-based n=1					
			40.9 (SD 8.3) 40.0	37.3 (SD 7.0) 37.0	*t*=4.77 *P*<.001	530
**Education, n (%)**						
	Missing: clinic n=5; Web-based n=11					
		Below university	196 (49.6)	62 (43.1)	χ^2^=1.65 *P*=.20	1
		University	199 (50.4)	81 (56.3)		
**Ethnicity, n (%)**						
	Missing: clinic n=6; Web-based n=0					
		White	346 (87.8)	143 (92.9)	χ^2^=2.93 *P*=.087	1
		Non-white	48 (12.2)	11 (7.1)		
**Employment, n (%)**						
	Missing: clinic n=22; Web-based n=18					
		Not in employment	151 (39.9)	37 (27.2)	χ^2^=7.00 *P*=.008	1
		In employment	227 (60.1)	99 (72.8)		
**Current ART** ^a^ **use, n (%)**						
	Missing: clinic n=9; Web-based n=0					
		Not on ART	125 (32.0)	64 (41.6)	χ^2^=4.49 *P*=.034	1
		On ART	266 (68.0)	90 (58.4)		
**MSAS** ^b^ **Global, mean (SD) median**						
	Missing: clinic n=0; Web-based n=12					
			1.15 (SD 0.79) 1.12	1.25 (SD 0.86) 1.23	*t*=−1.29 *P*=.20	529
**MSAS Physical, mean (SD) median**						
	Missing: clinic n=0; Web-based n=17					
			0.78 (SD 0.65) 0.73	0.70 (SD 0.74) 0.47	*t*=1.19 *P*=.23	517
**MSAS Psychological, mean (SD) median**						
	Missing: clinic n=0; Web-based n=4					
			1.36 (0.96) 1.33	1.61 (SD 1.09) 1.65	*t*=−2.69 *P*=.007	522
**MSAS Global (tertile groups), n (%)**						
	<0.72		137 (34.3)	45 (31.7)		
	0.72-1.52		136 (34.0)	45 (31.7)		
	>1.52		127 (31.8)	52 (36.6)	χ^2^(trend)=0.87 *P*=.35	2
**MSAS Physical (tertile groups), n (%)**						
	<0.27		131 (32.8)	55 (40.1)		
	0.27-1.0		126 (31.5)	47 (34.3)		
	>1.0		143 (35.8)	35 (25.5)	χ^2^(trend)=4.66 *P*=.031	2
**MSAS Psychological (tertile groups), n (%)**						
	<0.87		139 (34.8)	47 (31.3)		
	0.87-1.87		149 (37.3)	42 (28.0)		
	>1.87		112 (28.0)	61 (40.7)	χ^2^(trend)=4.32 *P*=.038	2

^a^ART: antiretroviral therapy

^b^MSAS: Memorial Symptom Assessment Scale

**Table 2:** Multivariable logistic analysis with (1) physical, and (2) psychological symptoms as independent variable, predicting upper tertile (ie, worst) symptom burden score.

		Physical symptoms as dependent variable (n=482)			Psychological symptoms as dependent variable (n=495)		
		Adjusted odds ratio (95% CI)		*P* value	Adjusted odds ratio (95% CI)		*P* value
**Setting**							
	Clinic^a^	1			1		
	Web-based	0.81 (0.50-1.30)	*P*=.38		2.20 (1.41-3.44)	*P*=.001	
**Age (years)**		1.00 (0.97-1.02)	*P*=.91		0.98 (0.95-1.00)	*P*=.98	
**Ethnicity**							
	Non-white^a^	1			1		
	White	1.40 (0.73-2.69)	*P*=.30		1.22 (0.64-2.24)	*P*=.54	
**Employment**							
	Not employed^a^	1			1		
	Employed	0.41 (0.28-0.62)	*P*<.001		0.32 (0.21-0.49)	*P*<.001	
**ART** ^b^ **use**							
	No^a^	1			1		
	Yes	1.39 (0.90-2.15)	*P*=.13		1.20 (0.78-1.85)	*P*=.40	

^a^reference category

^b^ART: antiretroviral therapy

## Discussion

### Principal Findings

Our data have revealed a number of differences between samples recruited via the Web and in clinic settings, in terms of both demographics and self-report psychological and physical symptom burden. First, as has been found with behavioral studies of gay men, the Web-based sample was younger than the clinic sample. Second, we found that those recruited via the Web were more likely to be in employment, which may reflect the costs associated with Internet connectivity. It may also be that those in employment face greater challenges in attending clinics regularly, or that the clinic sample had a higher proportion of participants with health problems linked to not being employed. Third, the clinic sample was more likely to be on ART. This may be because clinic sampling may under-sample those with poor or erratic attendance who are not on treatment or those with mild immunosuppression not yet on treatment and who feel less need to attend for care. In terms of the self-report symptom burden, participants in the Web-based sample were less likely to have high physical symptom burden; this difference was largely explained by the differences between the samples in demographic characteristics. However, the Web-based sample was more likely to have higher psychological symptom burden; this difference was not attenuated in adjusted analysis. There are several potential explanations for this latter finding. Psychological problems are more prevalent among HIV-infected populations (even among those on ART [21]) than the general population and those with other long-term conditions. It may be that the Web-based sample was actively seeking resources via the Web regarding the problems associated with living with HIV, and therefore accessed the survey materials. If the Web-based sample is, as we hypothesized above, less engaged with clinical services, this may be because their poorer mental health prevents them from attending regular care. Alternatively, it is possible that participants were more likely to report their psychological symptoms in the Web-based study due to a greater perceived anonymity of this study, or that those recruited via clinics are receiving better psychological care. In terms of intervention, while Web-based recruitment to intervention may enable those with worse psychological distress to be introduced to studies, it does not imply acceptability of Web-based interventions nor that those recruited in clinics may be more or less likely to take up Web-based interventions.

Our findings that the Web-based sample was statistically significantly younger and more likely to be employed are in line with previous studies [5,7]. However, our finding that they are also more likely to be in treatment and have lower psychological symptom burden is entirely new.

### Limitations

There are a number of limitations to our study. First, although we were able to compare a well-defined population by analyzing data from only those men in the Web-based survey with a London postal code to those accessing care at a London clinic, we note that men may travel to access care. Second, although we had a high response rate for the clinic survey (86%), we do not have a response rate for the Web-based survey.

For future research, we recommend that Web-based recruitment should include collection of the demographic variables that we note differed between samples: age, ART use, and employment. The effect of adjustment for these factors in analyses can then be examined. In addition, we recognize that each recruitment method may bring inherent sampling bias, with clinic populations differing by geographical populations served and reflecting those accessing regular medical care, and Web-based sampling recruiting those with greater Internet access and identifying survey materials through specific searches and contact with specific websites. Furthermore, our Web-based sampling did not allow specification of a sampling frame.

### Conclusions

Patient-reported symptom data can feasibly be collected though Web-based recruitment as well as through clinic-based questionnaire studies. There may be some specific advantages of Web-based studies when investigating stigmatized problems such as psychological burden of HIV disease, where social desirability may bias traditional face-to-face recruitment methods. We conclude that the Web-based sample had a higher psychological symptom burden (but not physical symptom burden) and that therefore they differ clinically from the sample recruited in clinics. This offers great opportunity to recruit people with HIV to interventions to improve mental health, which is a highly prevent and burdensome problem in this population.

## Abbreviations

ART: antiretroviral therapy
HIV: human immunodeficiency virus
MSM: men who have sex with men
MSAS-SF: Memorial Symptom Assessment Scale−Short Form
MSAS-PHYS: Memorial Symptom Assessment Scale−Physical Distress Subscale
MSAS-PSY: Memorial Symptom Assessment Scale−Psychological Distress Subscale
MSAS-GDI: Memorial Symptom Assessment Scale−Global Distress Subscale
